# Neural Mechanisms of Neuroticism: Large-Scale Brain Networks, Developmental Trajectories, and Translational Implications

**DOI:** 10.3390/brainsci16060610

**Published:** 2026-06-04

**Authors:** Xingye Ren, Lijuan An, Ning Jia, Kexin Lv, Zhanling Cui

**Affiliations:** Department of Psychology, Hebei Normal University, Shijiazhuang 050024, China; renxingye2021@163.com (X.R.); alj@hebtu.edu.cn (L.A.); jianing@hebtu.edu.cn (N.J.); 17761589217@163.com (K.L.)

**Keywords:** neuroticism, neural mechanism, multimodal neuroimaging, lifespan trajectory, amygdala, prefrontal cortex, default mode network, neural intervention

## Abstract

**Highlights:**

**What are the main findings?**

- Neuroticism is characterized by dysregulation of three large-scale brain circuits: hyperactive amygdala (emotion generation), hypoactive prefrontal cortex (cognitive control), and hyperconnected default mode network (self-referential rumination).
- A lifespan trajectory of Limbic Sensitivity → Regulatory Strain → Prefrontal Decline describes the dynamic evolution of neural vulnerability across adolescence, middle age, and old age.

**What are the implications of the main findings?**

- The Emotion–Cognition Imbalance Model identifies amygdala-prefrontal-DMN circuits as candidate neural targets for early intervention in affective disorders.
- The demonstrated malleability of neuroticism-related circuits through neuromodulation and behavioral interventions challenges the traditional view of neuroticism as a fixed trait.

**Abstract:**

Neuroticism, a Big Five trait characterized by emotional instability and susceptibility to negative affect, is a robust transdiagnostic predictor for the onset, severity, and persistence of anxiety disorders, major depressive disorder (MDD), and other affective conditions. Recent advances in functional magnetic resonance imaging (fMRI) techniques—including resting-state fMRI, multimodal neuroimaging, and their integration with machine learning—have enabled multi-perspective investigations into the neural substrates of neuroticism. Current research in this field primarily follows three complementary approaches: cross-sectional studies identifying key brain regions for emotional processing and cognitive control (e.g., amygdala (AMG), prefrontal cortex); longitudinal studies capturing neural mechanisms evolution across adolescence, middle age, and old age to elucidate relationships between neuroticism and brain plasticity; and intervention studies exploring plastic pathways for reshaping the neural representations of neuroticism, challenging the classic “trait stability” paradigm. This review synthesizes recent progress in the cognitive neuroscience of neuroticism across these three approaches, proposes a unified emotion-cognition neural model centered on the AMG-prefrontal-default mode network circuit, and outlines a hypothesized lifespan trajectory of Limbic Sensitivity → Regulatory Strain → Prefrontal Decline. While accumulated evidence broadly supports the cross-sectional and interventional pillars of this framework, the lifespan trajectory remains a theoretically informed working model requiring further longitudinal validation. The field still faces critical limitations, including small effect sizes, methodological heterogeneity, and unresolved questions regarding causality and circuit specificity. This review aims to provide a conceptual integration of existing findings, identify key uncertainties, and propose evidence-based future directions. We further link the proposed neural model to clinical phenotypic characteristics of high neuroticism and discuss its implications for targeted neural interventions, thereby advancing our understanding of the biological basis of neuroticism and providing a theoretical framework for prevention and intervention in neuroticism-related affective disorders.

## 1. Introduction

A primary objective of contemporary personality and cognitive neuroscience is to elucidate the neurobiological underpinnings of stable personality traits and their links to mental health outcomes [1]. Neuroticism is defined by a pronounced and persistent susceptibility to negative affect, emotional instability, and a low tolerance for aversive or stressful stimuli [2]. Among the canonical dimensions of the Big Five personality model, neuroticism has garnered intense scrutiny for its profound clinical relevance. Longitudinal and meta-analytic studies have confirmed that neuroticism is closely associated with the onset, chronicity, and recurrence of these affective conditions, with trait severity directly predicting poor treatment response and long-term functional impairment [3,4]. The critical scientific question is no longer whether neuroticism confers clinical risk, but how this vulnerability is instantiated at the level of neural systems, and how these mechanisms can be targeted for clinical intervention.

Historically, Hans Eysenck’s foundational factorial work identified Neuroticism as a major personality dimension distinct from Extraversion, and he proposed that individual differences in limbic system reactivity underlay variation in trait emotionality [5,6]. These early psychometric and biological theories laid the groundwork for later neuroimaging investigations.

An established convergent theoretical framework attributes high neuroticism to a core imbalance between hyperactive bottom-up emotional circuits and hypoactive top-down cognitive control systems [7,8]. This Emotion–Cognition Imbalance Model posits that the brain of individuals with high neuroticism is characterized by overactivation of the limbic and salience networks, which is inadequately mitigated by prefrontal executive control circuits [9]. Notably, this neural imbalance is not static; it is increasingly understood through a developmental lens that accounts for dynamic changes across the human lifespan [10,11]. This has led to the proposal of a theoretically informed Limbic Sensitivity–Regulatory Strain–Prefrontal Decline Lifespan Trajectory, which conceptualizes how this neural imbalance may originate in early life, manifest as acute clinical vulnerability in adolescence and early adulthood, and be amplified by age-related neural degeneration in later life [10,12].

Early research on the neurobiology of neuroticism relied predominantly on psychometric tools and behavioral observation, treating the brain as a “black box” and thereby limiting researchers’ ability to probe underlying neural mechanisms [13]. It should be noted, however, that before the advent of modern neuroimaging, psychophysiological studies using electroencephalography (EEG), event-related potentials (ERPs), electrodermal activity, and heart rate variability had already linked neuroticism to heightened cortical arousal, attentional bias to threat, and autonomic lability [14]. The advent of noninvasive neuroimaging techniques, including structural MRI (sMRI) and functional magnetic resonance imaging (fMRI), revolutionized the field, shifting the focus from static regional localization to the dynamic interactions of large-scale brain circuits [10]. Contemporary research has further expanded to include three core methodological pillars: network connectivity analysis via diffusion tensor imaging (DTI) and rs-fMRI, lifespan developmental tracking via longitudinal designs, and causal neural modulation via interventional studies [11,15]. These advances have established an integrated research architecture that encompasses stable brain structure, dynamic neural function, developmental context, and experimental modulation.

However, critical gaps remain in the field. First, existing studies have not fully established causal links between identified neural circuit abnormalities and the clinical phenotypic expression of neuroticism. Second, the dynamic evolution of these neural mechanisms across the lifespan has not been systematically integrated into a unified developmental framework. Third, the translational potential of basic neuroscience findings for clinical intervention remains under-explored. This review addresses these gaps by synthesizing empirical evidence from cross-sectional, longitudinal, and interventional studies to construct a unified emotion-cognition neural model (the Emotion-Cognition Imbalance Model) of neuroticism. We critically discuss the key brain circuits underlying the neurotic phenotype, present the proposed lifespan trajectory as a working model for future empirical testing, and address methodological challenges and future research directions. This review emphasizes the translational value of basic neuroscience findings, aiming to bridge the gap between personality neuroscience research and clinical practice for neuroticism-related affective disorders.

### Review Scope and Methodology

This article presents a narrative review that synthesizes empirical and theoretical advances in the cognitive neuroscience of neuroticism. Rather than attempting exhaustive coverage, we adopt a theoretically driven integrative approach, focusing on cross-sectional, longitudinal, and interventional studies that inform the Emotion-Cognition Imbalance Model and its developmental extensions. Literature was identified through systematic searches of PubMed, Web of Science, PsycINFO, and Google Scholar using combinations of the following keywords: “neuroticism,” “neurotic,” “personality neuroscience,” “amygdala,” “prefrontal cortex,” “default mode network,” “fMRI,” “resting-state fMRI,” “DTI,” “longitudinal,” “lifespan,” “neurofeedback,” “rTMS,” “tDCS,” and “emotion regulation.” The search period covered publications from 2001 to 2025, with priority given to peer-reviewed empirical studies, meta-analyses, and theoretical reviews published in English. Inclusion criteria were: (1) studies using multimodal neuroimaging (sMRI, fMRI, rs-fMRI, DTI, EEG/MEG) to examine neural correlates of neuroticism or its sub-facets; (2) longitudinal designs tracking neural or personality changes over time; (3) interventional studies (neurofeedback, neuromodulation, behavioral training) targeting neuroticism-related circuits. Exclusion criteria included: case reports with sample sizes below 10, non-human animal studies, and studies that did not report neuroimaging data. Because this is a narrative review, we do not claim exhaustive coverage; rather, we aim to integrate converging lines of evidence to propose a coherent working model and identify critical gaps for future research.

## 2. Core Neural Circuits Underlying Neuroticism

A large body of neuroimaging research suggests that the emotional instability and regulatory deficits characteristic of neuroticism are not caused by focal dysfunction in a single brain region, but rather by the dysregulation of three interconnected large–scale neural circuits–the emotion–generative network, cognitive–regulatory network, and self–referential (default mode) network [9,16]. This tripartite circuit dysregulation is consistent with the Emotion–Cognition Imbalance Model and may constitute a central neurobiological substrate of the neurotic phenotype. However, it is important to note that other large-scale networks, including the salience network and frontoparietal control network (FPCN), also contribute to emotional and cognitive regulation, and their roles are increasingly recognized (see Section 2.4).

### 2.1. Hyper-Responsive Emotion-Generative Network

The emotion-generative network is a bottom-up system that mediates threat detection, salience processing, and negative affect generation, centered on the amygdala (AMG), with key contributions from the anterior cingulate cortex (ACC) and insula (INS) [7,17]. In individuals with high neuroticism, this network often exhibits a lower activation threshold and prolonged response to aversive, ambiguous, or even neutral stimuli, providing a neural correlate for heightened stress sensitivity and persistent negative affect [7,18].

Structurally, high neuroticism has been linked to a complex pattern of AMG morphometric alterations, including cortical thinning and reduced gray matter volume [19,20]. Functionally, task-based fMRI (tb-fMRI) studies consistently demonstrate exaggerated AMG activation in highly neurotic individuals during exposure to negative emotional stimuli, even when the stimuli are presented subliminally [21,22]. This structural and functional hyper-reactivity may prime the brain for threat detection, amplifying emotional responses to negative cues and contributing to the persistent negative affect that defines the neurotic phenotype [23,24].

Notably, the hyper-responsiveness of this network is not limited to the AMG. The ACC, a key region for conflict monitoring and error detection, shows altered activation in response to emotional conflict in high neuroticism, while the INS, which mediates interoceptive awareness and subjective feeling states, exhibits hyper-connectivity with the AMG, which may prolong the duration of negative emotional states [10,25]. Together, these alterations contribute to a bottom-up system that is hypersensitive to threat and prone to sustained negative affect generation, which may be further amplified by deficits in the top-down regulatory system [25].

### 2.2. Hypo-Functional Cognitive-Regulatory Network

The cognitive-regulatory network is a top-down system responsible for emotion regulation, attentional deployment, and inhibitory control, predominantly composed of the lateral and medial prefrontal cortex (PFC), particularly the dorsolateral PFC (DLPFC) and ventromedial PFC [26]. This network is the primary neural substrate for modulating the activity of the limbic emotion-generative system, and its reduced efficiency is a hallmark of high neuroticism [9,27].

Convergent findings indicate that high neuroticism is associated with insufficient recruitment and inefficient functioning of the PFC during negative emotion regulation [21,27]. When confronted with aversive stimuli that require active regulatory control, the PFC of highly neurotic individuals shows diminished activation precisely when it is most needed, which may fail to effectively dampen AMG hyper-reactivity [21,22]. This functional deficit is further supported by structural connectivity evidence: DTI studies have revealed reduced white matter integrity of the uncinate fasciculus, the primary structural pathway connecting the PFC to the AMG and other temporal lobe limbic structures [28]. This degraded structural connectivity may create a functional disconnect between the top-down regulatory system and the bottom-up emotion-generative system, which is consistent with the core mechanistic tenet of the Emotion-Cognition Imbalance Model [20,21].

From a clinical perspective, prefrontal hypo-function directly maps onto the core phenotypic features of high neuroticism: impaired emotion regulation, poor impulse control, and an inability to disengage from negative emotional states [9,27]. The persistent failure of prefrontal regulatory control may further reinforce the hyper-reactivity of the limbic system, potentially creating a self-sustaining cycle of emotional dysregulation that may contribute to the onset and maintenance of affective disorders.

### 2.3. Dysregulated Self-Referential Network (Default Mode Network)

The default mode network (DMN) is a large-scale brain network comprising the medial PFC, posterior cingulate cortex, and precuneus (PCu), which mediates self-referential thought, introspection, rumination, autobiographical memory, and mind-wandering [29]. Its dysregulation is thought to underlie the cognitive component of neuroticism, including the trait’s characteristic tendencies toward worry, rumination, and negative self-appraisal [29,30].

Structurally, high neuroticism coincides with increased gray matter volume in core DMN hubs, particularly the PCu, a region closely linked to self-referential processing and autobiographical memory [31]. Functionally, rs-fMRI studies have demonstrated markedly enhanced intrinsic connectivity within the DMN in highly neurotic individuals, indicating a neural system that is prone to becoming “stuck” in self-referential thought loops [29]. Most critically, the DMN exhibits strengthened functional coupling with the AMG in high neuroticism [30]. This aberrant DMN-AMG connectivity may constitute a powerful reciprocal mechanism: self-referential rumination may fuel AMG-mediated negative affect, and heightened negative affect in turn amplifies DMN-mediated rumination, perpetuating a self-sustaining cycle of dysphoric mood and distress [9,30].

Clinically, this DMN dysregulation directly corresponds to the persistent worry, rumination, and negative self-evaluation that are core features of high neuroticism, and are key maintaining factors for depression and anxiety disorders [29,30]. It is important to acknowledge that the DMN also supports adaptive functions such as autobiographical memory retrieval and self-projection into the future; its dysregulation in neuroticism reflects a shift toward maladaptive, repetitive self-focus rather than a global network dysfunction [29].

### 2.4. Contributions from the Salience and Frontoparietal Control Networks

While the AMG-PFC-DMN circuit constitutes the dominant architecture in current neuroticism research, accumulating evidence suggests that the salience network (SN) and FPCN also play critical, albeit less systematically studied, roles. The SN, anchored in the dorsal ACC and anterior INS, is responsible for detecting behaviorally relevant stimuli and initiating appropriate network switching between the DMN and central executive networks [32]. In high neuroticism, SN hyper-reactivity may contribute to exaggerated detection of threat-related interoceptive and exteroceptive signals, thereby amplifying bottom-up emotional drive [18]. Concurrently, the FPCN, encompassing the DLPFC and inferior parietal lobule (IPL), supports adaptive cognitive control and flexible emotion regulation strategies [26]. Reduced FPCN connectivity and inefficient cross-network coordination between the FPCN and limbic structures have been observed in individuals with high negative affect, suggesting that deficits in dynamic network switching—rather than merely static PFC hypo-activation—may underlie regulatory failure [9,16]. Integrating these networks into the tripartite model provides a more comprehensive systems-level account of neuroticism, although further research is needed to clarify their distinct and interactive contributions relative to the core AMG-PFC-DMN circuit.

### 2.5. Heterogeneity of the Neurotic Phenotype: Sub-Facet Considerations

Neuroticism is not a unitary construct; it comprises distinct sub-facets, including anxiety, depressive affect, anger/hostility, self-consciousness, impulsiveness, and vulnerability [2]. Emerging evidence suggests that these sub-facets may map differentially onto the neural circuits described above. For example, the anxiety facet may be more strongly linked to AMG hyper-reactivity and SN dysfunction, whereas the depressive affect and self-consciousness facets may show stronger associations with DMN hyper-connectivity and rumination-related circuitry [29,30]. The anger facet, by contrast, may involve altered connectivity between the AMG and orbitofrontal regions implicated in reactive aggression regulation. Most existing neuroimaging studies treat neuroticism as a composite score, which may obscure sub-facet-specific neural signatures and limit the precision of personalized intervention. Future research should employ facet-level analyses to disentangle the distinct neural substrates of neuroticism’s components and to test whether sub-facet profiles predict differential treatment responses to targeted neuromodulation or behavioral interventions.

## 3. Three Complementary Research Approaches to Neurotic Neural Mechanisms

Contemporary research on the neural basis of neuroticism has evolved along three complementary methodological pathways, which together build a comprehensive understanding of the trait—from static neural correlates to dynamic lifespan evolution and causal plastic modulation. All three approaches are grounded in multimodal neuroimaging techniques, which are the core methodological foundation of modern personality neuroscience [10,15].

### 3.1. Cross-Sectional Studies: Static Neural Correlates of Neuroticism

Cross-sectional studies provide the foundational neuroanatomical and functional blueprint of neuroticism, offering a snapshot of the trait’s neural correlates at a single time point [9,10]. These studies use sMRI, tb-fMRI, rs-fMRI, and DTI to map structural and functional alterations in the tripartite neural circuits outlined above, and have established the core empirical basis of the Emotion–Cognition Imbalance Model.

Convergent cross-sectional findings have confirmed three core neural signatures of high neuroticism: (1) AMG structural abnormalities and functional hyper-reactivity during negative emotional processing [19,21]; (2) PFC hypo-activation during emotion regulation and reduced structural connectivity between the PFC and limbic system [21,28]; and (3) DMN hyper-connectivity and strengthened functional coupling between the DMN and AMG [29,30]. These findings have been replicated across multiple independent cohorts, ethnic groups, and age ranges, providing a robust and consistent map of the structural and functional brain architecture associated with neuroticism [9,16].

Nevertheless, the cross-sectional literature is not without inconsistencies. For instance, some studies report increased AMG volume in high neuroticism, whereas others find reduced volume or no significant association [19,20]. Similarly, DMN connectivity findings vary depending on whether connectivity is measured during rest or task conditions, and whether global signal regression is applied [29]. These discrepancies likely reflect heterogeneity in sample characteristics (age, sex, clinical status), imaging protocols (scanner field strength, preprocessing pipelines), and analytic strategies (seed-based vs. independent component analysis). Such methodological variability underscores the need for harmonized acquisition protocols and standardized preprocessing workflows to improve reproducibility across studies.

The inherent correlational nature of cross-sectional designs limits their ability to address two critical questions: first, whether the identified neural alterations are a cause or a consequence of high neuroticism; and second, how these neural signatures emerge, stabilize, or evolve over the lifespan [10,11]. These limitations are addressed by longitudinal studies, which add a critical temporal dimension to our understanding of neuroticism’s neural mechanisms.

### 3.2. Longitudinal Studies: Dynamic Lifespan Trajectory of Neurotic Neural Dysregulation

**Figure 1:**
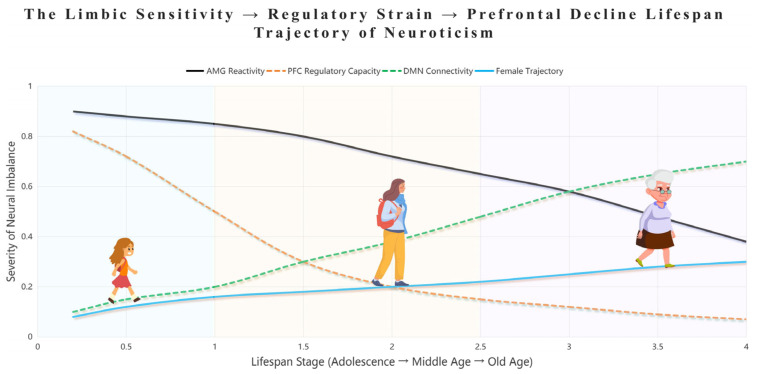
Hypothesized lifespan trajectory of neuroticism: limbic sensitivity → regulatory strain → prefrontal decline. Horizontal axis: lifespan stages (Adolescence, Middle Age, Old Age); vertical axis: neural imbalance severity (0~1.0, higher = more severe dysregulation). Black solid line (AMG reactivity): high in adolescence, decreasing with lifespan; Orange dashed line (prefrontal regulatory capacity): decreases faster, with a steeper slope. Green dashed line (DMN connectivity): rises in adolescence, the increase gradually slows in middle age, reaching a peak in old age; Blue solid line (female-specific): steadily increases with lifespan. Note: This figure presents a theoretically informed working model derived from converging cross-sectional meta-analytic trends and normative developmental neuroscience. Direct longitudinal neuroimaging evidence tracking these exact parametric trajectories within neuroticism-specific cohorts remains limited, and the curve shapes represent hypothesized dynamics awaiting empirical validation.

Longitudinal studies address the limitations of cross-sectional designs by tracking neural and behavioral changes within the same individuals over extended periods, capturing the dynamic plasticity of the brain across the human lifespan [10,11]. These studies provide essential empirical foundations for testing developmental hypotheses. In this section, we present the Limbic Sensitivity → Regulatory Strain → Prefrontal Decline lifespan trajectory as a theoretically informed working model that integrates available longitudinal evidence with developmental neuroscience principles. We explicitly distinguish between components that are directly supported by existing longitudinal data and those that remain theoretical extensions requiring future empirical validation. It is important to emphasize that this trajectory represents a theoretical synthesis of cross-sectional and normative developmental data; direct within-person longitudinal evidence across all three stages is not yet available (Figure 1).

#### 3.2.1. Adolescence: Limbic Sensitivity

Adolescence is a critical period for the consolidation of neuroticism’s neural signature, driven by normative developmental asynchrony between the limbic system and PFC [33]. During this period, the limbic emotion-generative system matures early, while the PFC regulatory network undergoes delayed structural and functional maturation, creating a temporary maturational gap in emotional regulation capacity [33]. In individuals with high neuroticism, this transient developmental imbalance may be exaggerated and reinforced, potentially consolidating the foundational neurophenotype that we hypothesize characterizes the “Limbic Sensitivity” stage.

At the neural level, this stage is characterized by three key alterations that are supported by converging cross-sectional and limited longitudinal evidence: (1) AMG enlargement, which is likely to lower the threshold for threat detection and amplifiy emotional responses to negative social cues [34]; (2) reduced ACC gray matter density, which might impair conflict monitoring and the initiation of corrective regulatory action, hindering the restoration of emotional homeostasis [34]; and (3) hyper-connectivity between the AMG and INS, which could create a reverberating circuit that prolongs anxious feelings and consolidates negative affect long after the initial trigger has passed [25]. Early deviations in frontolimbic white matter tracts further compromise the structural foundation for future top-down control, potentially setting the stage for the subsequent stages of the trajectory [35,36].

Testable predictions derived from this model include: (a) individuals with high neuroticism in early adolescence will show steeper AMG volume growth and weaker frontolimbic white matter maturation trajectories compared to low-neuroticism peers; (b) the magnitude of adolescent limbic-PFC connectivity imbalance will predict first-onset anxiety or depressive episodes within 5–10 years; and (c) early neuromodulation or behavioral interventions targeting frontolimbic connectivity could potentially alter developmental trajectories toward more adaptive patterns and may reduce later psychopathology risk.

#### 3.2.2. Middle Age: Regulatory Strain

Middle age marks the transition to the “Regulatory Strain” stage, characterized by the progressive exhaustion of PFC compensatory mechanisms [10,36]. Although the PFC reaches its peak structural and functional maturity during this life stage, it faces a persistent triple compensatory burden that may originate from the adolescent-onset deficits: (1) progressive microstructural decay of the frontolimbic white matter tracts that mediate top-down inhibitory control [10]; (2) chronic metabolic strain from sustained hypothalamic–pituitary–adrenal (HPA) axis overactivation and high allostatic load, which may be driven by persistent limbic hyper-reactivity [35,36]; and (3) the unremitting demand to modulate the hyperactive emotion-generative system over decades.

This accumulated burden may progressively erode emotion regulation efficiency, weaken PFC inhibitory signaling, and shift from a strained but functional dynamic equilibrium toward a state of chronic neural dysregulation [10,37]. Clinically, this stage marks increased susceptibility to first-onset affective disorders and psychosomatic depletion (see Table 1) [37,38]. Notably, this stage is also when sex differences in neuroticism’s neural trajectory become most pronounced: females may exhibit faster PFC aging and smaller functional reserves, potentially leading to an accelerated transition to regulatory strain and earlier onset of functional impairment [39]. These sex differences are likely moderated by multiple factors, including hormonal fluctuations (e.g., estrogens modulating prefrontal dopaminergic tone and HPA axis reactivity), genetic vulnerabilities (e.g., FKBP5 and BDNF polymorphisms interacting with sex chromosomes), and sociocultural stressors (e.g., gender-role-related chronic psychosocial burden) [39]. It is important to emphasize that sex differences in neural aging are context-dependent and moderated by stress exposure, reproductive history, and cultural factors; they should not be interpreted as uniform, biologically deterministic trajectories.

#### 3.2.3. Old Age: Prefrontal Decline

Old age represents the final stage of the hypothesized trajectory: “Prefrontal Decline”. Normative age-related prefrontal degeneration may be exacerbated by decades of chronic stress and compensatory strain, potentially contributing to progressive structural atrophy that is difficult to reverse, particularly in the orbitofrontal cortex (OFC), a region critical for emotion regulation and decision-making [40]. This sustained atrophy may deplete the brain’s neural and cognitive reserves, triggering a cascade of cognitive control network failure [12].

High neuroticism may further amplify this process (Table 1): the trait’s characteristic exaggerated threat perception and chronic stress may accelerate prefrontal volume loss, while the already compromised frontolimbic regulatory circuitry may undergo progressive decompensation [13,40]. Clinically, this stage manifests as geriatric affective-cognitive comorbidity and treatment resistance (see Table 1) [41]. This stage is consistent with the view that high neuroticism is not only a risk factor for affective disorders, but may also act as a contributor to pathological brain aging, although causal directionality remains to be established.

### 3.3. Interventional Studies: Causal Neural Plasticity of Neuroticism

Interventional studies are the most compelling approach for establishing causal relationships between neural circuit dysregulation and neuroticism [11,14]. By experimentally modulating the key nodes and circuits identified in cross-sectional and longitudinal studies, these studies test the plasticity of the neurotic brain, provide preliminary support for the core tenets of the Emotion–Cognition Imbalance Model, and explore targeted clinical intervention pathways—bridging basic neuroscience research and clinical application. Current evidence-based interventions follow three core strategies, all of which have shown promising effects in reducing neurotic traits and improving emotional regulation capacity in proof-of-concept trials (see Table 2). However, most studies are small-scale, short-term, and lack active control conditions or multi-site replication; therefore, findings should be interpreted as preliminary evidence requiring validation through large-scale, preregistered randomized controlled trials (RCTs).

#### 3.3.1. Bolstering Prefrontal Cognitive Regulation

This strategy targets the DLPFC, the core hub of the cognitive-regulatory network, to enhance top-down control over the hyperactive limbic system. Excitatory rTMS to the left DLPFC is the most well-established intervention in this category: A clinical trial has shown that this approach increases DLPFC neural activity, strengthens frontolimbic regulatory connectivity, and significantly reduces neuroticism scores [42]. Real-time fMRI NFB training, which teaches individuals to voluntarily modulate their own brain activity, has further shown that strengthening functional connectivity between the left DLPFC and INS has been associated with improvements in emotion regulation and reductions during the intervention period and immediate follow-up [14,43]. However, these findings are based on small samples (typically *n* < 40) and short follow-up periods; whether these neural changes translate to long-term trait modification remains uncertain.

#### 3.3.2. Modulating Self-Referential Processing (DMN)

This strategy aims to dampen DMN hyper-connectivity and break the aberrant coupling that drives rumination and negative self-focus. Inhibitory tDCS over the IPL, a key DMN node for autobiographical memory and self-referential processing, has shown preliminary efficacy in reducing immersion in negative self-related memories and facilitate emotional disengagement from critical self-referential thoughts in highly neurotic individuals [44]. Theta-band NFB training targeting the IPL further improves the regulation of emotional memories and reduces rumination [45]. On a broader network level, mindfulness-based behavioral training, which is designed to reduce DMN hyperactivity and disengage automatic negative thought patterns, has been widely applied in clinical settings, with meta-analyses confirming its efficacy in reducing neuroticism, rumination, and anxiety symptoms [46,47]. Nevertheless, the heterogeneity of mindfulness protocols and the absence of active control conditions in many studies limit causal inference.

#### 3.3.3. Retuning Core Affective Hubs

This strategy targets the key interface regions between the emotion-generative and cognitive-regulatory systems, including the ACC and aINS, which mediate conflict monitoring and subjective emotional experience [32,48]. Cognitive reappraisal training, a core component of cognitive behavioral therapy (CBT), directly engages the ACC to enhance executive control over emotional responses and reduce the tendency to ruminate, with long-term follow-up studies showing that this training reduces neuroticism and lowers the risk of recurrent affective episodes [49]. Real-time fMRI NFB targeting the right aINS has further demonstrated that down-regulating activity in this region disrupts the specific neural pathway linking high neuroticism to depressive symptoms, potentially providing a highly targeted mechanism for intervention [15]. These findings remain preliminary, as most studies employ small, treatment-seeking samples and lack control for non-specific therapeutic factors.

Collectively, interventional studies provide preliminary evidence consistent with the plasticity of neuroticism’s neural mechanisms: this deeply ingrained personality trait may not be an immutable fixture of an individual’s neurobiology, but may be modified by targeted modulation of the tripartite core circuits. These findings not only provide preliminary causal support for the Emotion-Cognition Imbalance Model, but also lay the empirical foundation for the development of personalized, mechanism-based clinical interventions for high neuroticism and its related affective disorders.

## 4. Proposed Integrated Theoretical Framework

Building on the empirical evidence synthesized above, we propose a unified, dual-component integrated theoretical framework for the neural basis of neuroticism: the Emotion-Cognition Imbalance Neural Model and the Limbic Sensitivity → Regulatory Strain → Prefrontal Decline Lifespan Trajectory. This framework integrates static cross-sectional findings, dynamic longitudinal developmental data, and causal interventional evidence, providing a coherent, mechanistically informed working model of neuroticism’s neural underpinnings and their clinical implications.

At the core of the framework is the Emotion-Cognition Imbalance Neural Model, which synthesizes the cross-sectional and interventional evidence reviewed above. Rather than positing new regional mechanisms, this model integrates the three empirically supported circuit-level alterations detailed in Section 2—hyper-responsive limbic generation, hypo-functional prefrontal regulation, and dysregulated DMN-mediated self-referential processing—into a unified systems-level account. Within this model, the functional disconnect between bottom-up emotional drive and top-down regulatory capacity is hypothesized to constitute the primary mechanistic source of emotional instability in high neuroticism, whereas aberrant DMN–AMG coupling is posited to amplify and sustain this imbalance through recurrent self-referential rumination (see Section 2.1, Section 2.2 and Section 2.3 for circuit-specific evidence).

The second component of the framework is the lifespan trajectory, which describes the hypothesized dynamic evolution of this core neural imbalance across the human lifespan. The trajectory unfolds in three sequential, cascading stages: (1) adolescence, where developmental asynchrony between the limbic system and PFC may contribute to the consolidation of limbic sensitivity and the establishment of the core neurophenotype; (2) middle age, where decades of compensatory regulatory demand may contribute to progressive frontolimbic tract degradation, PFC metabolic exhaustion, and the transition to regulatory strain; (3) old age, where normative age-related neural degeneration may exacerbate pre-existing regulatory deficits, potentially contributing to progressive prefrontal decline that is difficult to reverse and accelerated pathological brain aging. Biological sex may serve as a critical moderator of this trajectory, with females—under the influence of hormonal, genetic, and sociocultural factors—potentially exhibiting an earlier onset, faster progression, and more severe phenotype across all three stages, offering a neurobiological working hypothesis for the well-documented sex disparities in neuroticism-related affective disorders.

This integrated framework addresses critical gaps in existing theoretical models: it unifies static and dynamic findings into a single coherent working model, offers testable hypotheses linking neural circuit dysregulation to clinical phenotypic expression, and provides a mechanistic account of neuroticism’s developmental trajectory across the lifespan. It also has potential clinical implications: the framework identifies specific, targetable neural nodes and circuits for intervention, and highlights the importance of early, developmentally tailored interventions to potentially prevent the progression of the pathological trajectory and reduce the risk of long-term mental health outcomes.

While the current framework primarily treats neuroticism as a unified construct, emerging evidence suggests that its sub-facets (e.g., anxiety, depressive affect, anger) may map differentially onto the tripartite circuits. Future refinements of the model should incorporate facet-level specificity.

## 5. Methodological Challenges and Limitations

Despite significant advances in the field, our understanding of neuroticism’s neural mechanisms is still limited by several persistent methodological and conceptual challenges, which define the critical frontiers for future research.

### 5.1. Causality and Directionality

The field has not yet fully established causal relationships between neural circuit alterations and neuroticism. Cross-sectional designs, which form the majority of existing research, can only identify correlational associations, not causal directionality [10,11]. Longitudinal studies, while providing a critical temporal dimension, are limited by practical constraints, including high sample attrition, insufficient follow-up durations, and a lack of multi-decade studies that can fully validate the proposed lifespan trajectory [10,11]. Interventional studies, the gold standard for establishing causality, are often limited by small sample sizes, short-term follow-up periods, and a lack of active control conditions, making it difficult to confirm whether intervention-induced neural changes are sustained over time and translate to long-term trait modification [14].

### 5.2. Spatial-Temporal Trade-Offs in Neuroimaging

Existing neuroimaging techniques have inherent spatial–temporal trade-offs that limit our ability to capture the dynamic neural interactions that underlie neuroticism [9,11]. fMRI offers excellent spatial localization, but operates on a timescale of seconds, which is too slow to capture the millisecond-scale dynamic interplay between the AMG and PFC that is central to emotion regulation [22,23]. In contrast, EEG and magnetoencephalography (MEG) provide superb temporal resolution, but have poor spatial precision, making it difficult to accurately localize signals from deep subcortical limbic structures that are core to the model [11]. Additionally, sMRI and DTI lack the sensitivity to detect subtle microstructural plastic changes in brain tissue that occur in response to emotion regulation or therapeutic intervention [11,15], limiting our understanding of neural plasticity at the microstructural level.

### 5.3. Multimodal Integration and Analytical Heterogeneity

The field lacks standardized analytical frameworks for integrating multimodal neuroimaging data [11,15]. Most existing studies analyze structural, functional, and connectivity data in isolation, leading to fragmented and occasionally conflicting interpretations of neuroticism’s complex neural architecture. For example, structural abnormalities identified by sMRI often do not align with functional changes detected by fMRI, and without integrated analysis, the mechanistic link between these alterations remains unclear. This fragmentation hinders the development of a comprehensive, systems-level model of the neurotic brain, and limits the identification of stable, cross-modal neural signatures of neuroticism that can be used for clinical risk stratification and intervention targeting. Furthermore, substantial variability in imaging protocols (scanner manufacturer, field strength, pulse sequence parameters), preprocessing pipelines (software versions, motion correction strategies, global signal regression), and statistical models (mass univariate vs. multivariate pattern analysis) contributes to low reproducibility and complicates cross-study comparison [11].

### 5.4. Phenotypic Heterogeneity and Publication Bias

Existing research has not fully accounted for the substantial heterogeneity of the neurotic phenotype. Neuroticism is a multifaceted trait, with distinct sub-facets (e.g., anxiety, depression, anger, self-consciousness) that may have distinct neural underpinnings (see Section 2.5). Most existing studies treat neuroticism as a unitary construct, which may mask important differences in the neural mechanisms of its sub-facets, and limit the development of personalized, sub-facet-specific interventions. Finally, the field may be subject to publication bias, with small studies reporting null or contradictory findings remaining unpublished, thereby inflating effect sizes in the published literature and distorting meta-analytic estimates [7,9]. Sample heterogeneity—spanning healthy volunteers, subclinical populations, and clinical patients—further complicates generalization across studies.

## 6. Future Research Directions

To address the above limitations and advance the field, future research on neuroticism’s neural mechanisms should focus on four core priority areas, aligned with the scope and mission of Brain Sciences to advance basic brain science research and its clinical translation.

First, future research should implement integrated longitudinal-interventional designs to establish stronger causal relationships and validate the proposed lifespan trajectory [10,11]. By combining prospective, multi-decade longitudinal tracking of large community cohorts with targeted neural intervention experiments, researchers can test whether modulating the core neural circuits of neuroticism alters the developmental course of the trait, and clarify the causal direction of the links between neural circuit dysregulation and clinical outcomes. These designs should also account for individual differences in neural baselines and trait sub-facets, laying the foundation for personalized intervention strategies tailored to an individual’s unique neurofunctional profile.

Second, future research should leverage advanced multimodal neuroimaging techniques to overcome the spatial-temporal trade-offs of single-modality imaging [11,12]. Simultaneous acquisition of fMRI-EEG/MEG data will provide high spatial resolution to localize key circuit nodes and high temporal resolution to track the millisecond-scale dynamic interactions between the AMG and PFC during emotion regulation, enabling a more precise understanding of the temporal dynamics of the core neural imbalance. In parallel, the development and application of high-resolution microstructural imaging techniques (e.g., neurite density imaging) will allow researchers to detect subtle microstructural plastic changes induced by therapeutic intervention, deepening our understanding of neural plasticity at the cellular level.

Third, future research should develop standardized multimodal data integration frameworks and leverage advanced machine learning methods to identify stable, cross-modal neural signatures of neuroticism [11,50]. Standardized workflows for integrating structural, functional, and connectivity data will enable a more comprehensive, systems-level understanding of the neurotic brain, resolving conflicting findings from isolated modality analyses. Advanced machine learning algorithms, including graph neural networks and deep learning, can be applied to mine large-scale multimodal neuroimaging datasets to extract robust, generalizable neural signatures of neuroticism, which can be used for clinical risk stratification, early identification of at-risk individuals, and prediction of intervention response.

Fourth, future research should prioritize the clinical translation of basic neuroscience findings, developing and validating personalized, mechanism-based interventions for high neuroticism [14,44]. Building on existing proof-of-concept studies, large-scale, multi-center, preregistered RCTs are needed to validate the long-term efficacy of combined intervention strategies (e.g., rTMS + NFB + CBT) for reducing neuroticism and preventing the onset of affective disorders. Additionally, research should explore novel intervention targets, including the specific thalamocortical circuits that have been implicated in emotional arousal regulation in recent Brain Sciences publications [23], and develop portable, non-invasive neuromodulation devices that can be deployed in clinical and community settings, expanding the accessibility of targeted interventions. Critically, intervention designs should stratify participants by neuroticism sub-facet profiles to test whether circuit-specific neuromodulation produces differential outcomes for anxiety-predominant versus rumination-predominant phenotypes.

Finally, future research should systematically investigate sex differences in the neural mechanisms of neuroticism, as well as the moderating effects of hormonal status, genetic polymorphisms, sociocultural context, and environmental stressors. This will enable the development of more equitable, tailored interventions that account for individual and group differences in neurobiological vulnerability, and will help clarify whether sex differences are universal or context-dependent.

## 7. Conclusions

This review synthesizes recent advances in the cognitive neuroscience of neuroticism across cross-sectional, longitudinal, and interventional research approaches, and proposes a unified integrated theoretical framework comprising the Emotion-Cognition Imbalance Neural Model and the Limbic Sensitivity → Regulatory Strain → Prefrontal Decline Lifespan Trajectory. The core of this framework is the dysregulation of three interconnected large-scale brain circuits: a hyper-responsive AMG-centered emotion-generative network, a hypo-functional PFC-centered cognitive-regulatory network, and a dysregulated DMN-mediated self-referential network. This tripartite circuit dysregulation may form a key neural substrate of the emotional instability and clinical vulnerability that define high neuroticism, with its dynamic evolution across the lifespan potentially shaped by developmental asynchrony, chronic compensatory strain, and age-related neural degeneration. Interventional studies have provided preliminary support for the plasticity of these neural mechanisms, offering initial evidence consistent with the model and a foundation for targeted clinical interventions.

Despite significant progress, the field still faces critical limitations, including unresolved causal relationships, inherent trade-offs in neuroimaging techniques, a lack of standardized multimodal data integration frameworks, insufficient accounting for phenotypic heterogeneity, and potential publication bias. Future research should address these limitations through integrated longitudinal-interventional designs, advanced multimodal neuroimaging techniques, standardized data integration workflows, and large-scale, preregistered clinical translation studies.

This review advances the field of personality neuroscience by providing a coherent, mechanistically informed working model of neuroticism’s neural underpinnings, and establishing a conceptual link between basic neuroscience findings and clinical practice. By deepening our understanding of the biological basis of neuroticism, this framework not only enhances our knowledge of individual differences in personality and emotional regulation, but also provides new insights for the early prevention, risk stratification, targeted intervention and treatment of neuroticism-related affective disorders. As neuroimaging and neuromodulation techniques continue to evolve, the study of neuroticism will remain a key frontier in personality neuroscience, with profound implications for mental health promotion and the treatment of affective disorders.

## Figures and Tables

**Table 1:** Core neural features and corresponding clinical phenotypes across the lifespan trajectory of neuroticism.

Lifespan Stage	Core Neural Feature	Clinical Phenotype
Adolescence (Limbic Sensitivity)	AMG enlargement, reduced ACC gray matter density, AMG-INS hyper-connectivity, frontolimbic white matter immaturity	Heightened social anxiety, emotional lability, stress intolerance, peer rejection sensitivity ♀
Middle Age (Regulatory Strain)	Progressive frontolimbic tract degradation, PFC metabolic depletion, sustained AMG hyper-reactivity, elevated allostatic load	Chronic fatigue, somatic symptoms, occupational burnout, persistent rumination, increased risk of first-onset MDD/anxiety ♀
Old Age (Prefrontal Decline)	OFC volume loss, widespread PFC gray matter atrophy, complete frontolimbic functional disconnect, depleted cognitive reserves	Geriatric depression/anxiety comorbidity, accelerated cognitive decline, impaired emotion regulation, poor treatment response ♀

Note: This table summarizes a theoretically informed working model. Female-specific phenotypic characteristics are suggested by sex difference research in neuroticism, though effect sizes are often modest and moderated by hormonal, genetic, and sociocultural factors.

**Table 2:** Summary of Targeted Neural Interventions for High Neuroticism.

Intervention Strategy	Target Brain Region/Circuit	Technical Approach	Core Neural Effect	Clinical/Behavioral Outcome	Evidence Level & Limitations
Bolstering Prefrontal Cognitive Regulation	Left DLPFC; DLPFC-INS functional connectivity	Excitatory repetitive TMS (rTMS); real-time fMRI-Neurofeedback (NFB) training	Enhanced DLPFC neural activity; strengthened frontolimbic regulatory connectivity	Reduced neuroticism scores; improved emotion regulation; alleviated depressive and anxiety symptoms	Preliminary: Single-site RCTs (*n* = 20–40); short follow-up (≤4 weeks); lack of active sham-controlled designs [42,43]
Modulating Self-Referential Processing (DMN)	IPL; DMN intrinsic connectivity; DMN-AMG coupling	Inhibitory transcranial direct current stimulation (tDCS); theta-band NFB training; mindfulness-based behavioral training	Reduced IPL activity for negative self-referential memories; dampened DMN hyper-connectivity; weakened DMN-AMG functional coupling	Reduced rumination and worry; diminished negative self-appraisal; disengagement from automatic negative thought patterns	Emerging: Small pilot studies (*n* = 15–30); heterogeneous mindfulness protocols; effect sizes small-to-moderate (Cohen’s d ≈ 0.3–0.6) [44,45,46,47]
Retuning Core Affective Hubs	Right anterior INS (aINS); ACC; emotion-regulatory interface circuits	real-time fMRI-NFB (down-regulation); cognitive reappraisal training	Reduced aINS activity for subjective negative affect; enhanced ACC conflict monitoring and executive control	Disrupted the neural pathway linking neuroticism to depressive symptoms; improved emotional conflict resolution; reduced persistent negative affect	Proof-of-concept: Pilot trials (*n* = 10–25); no long-term durability data; generalizability to subclinical populations unknown [15,32,48,49]

Note: All listed interventions have demonstrated efficacy in proof-of-concept or small-scale clinical trials. Large-scale, multi-center, preregistered RCTs with long-term follow-up are needed to validate clinical effectiveness, durability of effects, and generalizability across neuroticism sub-facets.

## Data Availability

No new data were created or analyzed in this study. Data sharing is not applicable to this article.

## Abbreviations

The following abbreviations are used in this manuscript:

ACC	Anterior Cingulate Cortex
aINS	Anterior Insula
AMG	Amygdala
CBT	Cognitive Behavioral Therapy
DLPFC	Dorsolateral Prefrontal Cortex
DMN	Default Mode Network
DTI	Diffusion Tensor Imaging
fMRI	Functional Magnetic Resonance Imaging
FPCN	Frontoparietal Control Network
HPA	Hypothalamic–Pituitary–Adrenal
INS	Insula
IPL	Inferior Parietal Lobule
MDD	Major Depressive Disorder
NFB	Neurofeedback
OFC	Orbitofrontal Cortex
PCu	Precuneus
PFC	Prefrontal Cortex
RCT	Randomized Controlled Trial
rs-fMRI	Resting-State Functional Magnetic Resonance Imaging
rTMS	Repetitive Transcranial Magnetic Stimulation
SN	Salience Networks
sMRI	Structural Magnetic Resonance Imaging
tb-fMRI	Task-Based Functional Magnetic Resonance Imaging
tDCS	Transcranial Direct Current Stimulation
TMS	Transcranial Magnetic Stimulation
